# Costs of Suppressing Emotional Sound and Countereffects of a Mindfulness Induction: An Experimental Analog of Tinnitus Impact

**DOI:** 10.1371/journal.pone.0064540

**Published:** 2013-05-10

**Authors:** Hugo Hesser, Peter Molander, Mikael Jungermann, Gerhard Andersson

**Affiliations:** 1 Department of Behavioural Sciences and Learning, Swedish Institute for Disability Research, Linköping University, Linköping, Sweden; 2 Department of Behavioural Sciences and Learning, Linköping University, Linköping, Sweden; 3 Psychiatry Section, Department of Clinical Neuroscience, Karolinska Institutet, Stockholm, Sweden; Goldsmiths, University of London, United Kingdom

## Abstract

Tinnitus is the experience of sounds without an appropriate external auditory source. These auditory sensations are intertwined with emotional and attentional processing. Drawing on theories of mental control, we predicted that suppressing an affectively negative sound mimicking the psychoacoustic features of tinnitus would result in decreased persistence in a mentally challenging task (mental arithmetic) that required participants to ignore the same sound, but that receiving a mindfulness exercise would reduce this effect. Normal hearing participants (*N* = 119) were instructed to suppress an affectively negative sound under cognitive load or were given no such instructions. Next, participants received either a mindfulness induction or an attention control task. Finally, all participants worked with mental arithmetic while exposed to the same sound. The length of time participants could persist in the second task served as the dependent variable. As hypothesized, results indicated that an auditory suppression rationale reduced time of persistence relative to no such rationale, and that a mindfulness induction counteracted this detrimental effect. The study may offer new insights into the mechanisms involved in the development of tinnitus interference. Implications are also discussed in the broader context of attention control strategies and the effects of emotional sound on task performance. The ironic processes of mental control may have an analog in the experience of sounds.

## Introduction

Despite our best efforts to engage in mental activities (e.g., reading, writing), sounds of relatively low intensity can force themselves on our awareness and interrupt what we are doing (c.f., irrelevant-sound effect; [1]). Unwanted and uncontrollable sounds may be particularly interfering when they evoke strong negative emotional reactions. At such moments, an individual may resort to mental control strategies, such as trying to avoid thinking about the sound, in an attempt to control behavior and minimize interference. Yet, as an analog to theories on mental control such strategies may backfire [2]. That is, suppression as effortful regulation strategy may prolong and intensify the experience of the sound, creating more interference over time. Drawing on theories of mental control [3], [4], the present study examined attentional strategies in the context of emotional sound that mimicked the psychoacoustic characteristics of tinnitus.

Tinnitus is the experience of sounds without any external auditory source [5]. The sounds may vary greatly but are generally conceived as ringing, hizzing or buzzing. These auditory sensations are often accompanied by hearing loss, and there is considerable overlap between tinnitus and other auditory-related problems, including hyperacusis (noise sensitivity) and dizziness/vertigo [5]. Tinnitus is common with prevalence rates of 10 to 15% in the general adult population [6], [7], and the incidence seems to be rising as due to increasing noise exposure and an aging population in the western part of the world [8]. The majority of those affected, however, do no conceive tinnitus as a source of great distress, but for a subsample of individuals, tinnitus sounds can trigger strong emotional reactions and seriously impact daily functioning [8], [9]. In particular, individuals who find tinnitus to be annoying often report concentration difficulties, an observation that parallel recent findings showing that cognitive ability is generally compromised in individuals with tinnitus [10], [11].

The role of psychological variables as a mediating link between sound perception and interference/distress was noted early [12]. Given this, psychological approaches were developed and tested at an early stage, and over the years behavioral and cognitive treatment techniques targeting the consequences of tinnitus have gained empirical support [13], [14]. More recently, neuroscience research has provided evidence suggesting that non-auditory brain regions subserving emotions and attention play an essential role in both tinnitus perception and interference [15], [16]. Thus, tinnitus is intertwined with emotional and attentional processing. However, to date the mechanisms by which tinnitus causes distress and interference are largely unknown. Still, certain theories posit that the ways in which the auditory stimuli is appraised, processed and attended to may play a pivotal role in the development and maintenance of tinnitus distress [16], [17], [18]. For example, Hallam et al. [18] suggested that a preoccupation with tinnitus would slow down the natural process of habituating to tinnitus. Unfortunately, Hallam el al. offered little details of the psychological processes that would produce such a preoccupation with tinnitus in the first place. In fact, there is a paucity of research on psychological processes involved in tinnitus perception and interference.

Theories on mental control, such as the Ironic Process Theory [2], [19], may provide a useful explanation for the underlying processes involved in the development and maintenance of tinnitus interference, in particular why certain individuals may become preoccupied with the sensation. The Ironic Process Theory suggests that efforts to control mental states to achieve a particular desired state of mind often result in effects diametrically opposite of the original intent. For example, the theory predicts that efforts aimed at suppressing a particular thought will ultimately fail and will give rise to increased occurrence of the target thought. Indeed, suppression – a term associated with expressive suppression, thought suppression, and experiential avoidance – is one of the most widely studied regulation-strategies of mental and emotional events [20], [21]. There is considerable evidence to suggest that these strategies are counterproductive when applied to emotions or thoughts, as they tend to maintain, or even paradoxically increase, the internal sensations that they are suppose to regulate, especially under mental load [2], [22].

More recent empirical studies have shown that this phenomenon has an analog in the domain of physical sensations, in particular in relation to pain [23], [24]. For example, Cioffi and Holloway [23] found that subjects instructed to suppress the experience of pain experienced slower recovery from a cold-pressor pain induction and rated a second somatic induction as more unpleasant as compared with subjects who were instructed to monitor physical sensations or to use distraction. Thus, it appears that attentional strategies specifically designed to remove a “forbidden” sensation or mental event from mind can produce negative consequences over time, exacerbating the very sensation that one was trying to avoid. Moreover, such a strategy may deplete mental resources and compromise goal-direct behavior. Indeed, mental and emotional control strategies have consistently shown to impact performance negatively in subsequent task requiring self-control (c.f., ego-depletion; [25], [26]). Studies have shown maladaptive behavioral consequences of suppression, including decreased persistence [25], behavioral control [27], and willingness to approach emotionally evocative tasks [28]. It is important to note that these effects are not observed directly when the suppression strategy is employed, but show up later, for example, when individuals abandon the attempt to suppress, are asked to revisit the suppressed target at a later time [22], or when they are engaged in subsequent tasks that require high-degree of self-control [25].

Counterintuitively, strategies that increase attention to interfering or aversive stimuli may in fact be a better way of facilitating adaptive responding. Sensory monitoring – which is to attend, in a neutral way, to discrete sensory aspects of a sensation – has in series of laboratory experiments shown to be an effective strategy to increase pain tolerance and reduce discomfort during noxious stimulation [29], [30]. However, the effect of monitoring is often moderated by length and intensity of the stimulus. That is, distraction often works better when the stressor is acute, whereas monitoring works better when the stimulus is persistent [29], [31].

Mindfulness is “a process of regulating attention in order to bring a quality of non-elaborative awareness to current experience and a quality of relating to one's experience within an orientation of curiosity, experiential openness, and acceptance.” ([32] p. 234). Defined in this way, mindfulness bares many similarities with sensory monitoring. Indeed, it has been associated with similar beneficial outcomes in laboratory settings [33], [34]. Mindfulness is often utilized in treatments as means to promote psychological acceptance, which connotes an active process of allowing internal reactions (physiological, emotional, or cognitive) to be experienced as they are without defense or control [3], [35]. Recent theories posit that such a stance undermines maladaptive strategies (e.g., suppression), and, thereby, reduces the harmful effects associated with control strategies (see, [36], for a review of different treatments promoting similar processes; see also [22]). A growing body of experimental work suggests that, when compared to approaches such as suppression, acceptance can be an adaptive strategy to increase tolerance of aversive stimuli and reduce negative affect (e.g., induced pain or anxiety; [28], [37], [38]). There is also tentative evidence to support that acceptance of emotions requires fewer resources than suppression and that it may therefore improve performance in tasks requiring self-control [39]. To sum, attending mindfully to sensations rather than suppressing them may, at least in certain circumstances, be a better approach to reduce distress and facilitate adaptive responding during aversive stimulation.

Despite its potential relevance for the effects of sound on performance in general and for tinnitus interference in particular, little experimental work has been conducted to examine the effects of the above-mentioned attentional strategies in the context of auditory stimuli. However, there is growing evidence for that these strategies are in fact of importance in the development and maintenance of tinnitus interference and distress. That is, correlational studies have provided initial support to the beneficial consequences of acceptance on the experience of tinnitus [40], [41], [42], and avoidance-based coping has found to be associated with increased tinnitus-related distress [43], [44]. Furthermore, psychological treatments that have incorporated mindfulness/acceptance related-techniques have recently been shown to be effective in treating distress and impact of tinnitus [45], [46], [47].

Yet, only a few experimental studies have been carried out on related processes and they have provided mixed findings. For example, Andersson et al. [48] compared instructions to suppress or to attend to thoughts about tinnitus with a control condition in participants with tinnitus. They found that, immediately following manipulation, instructions to suppress decreased thoughts about tinnitus whereas instructions to attend to tinnitus increased such thoughts. In another tinnitus study the effects of suppression and acceptance strategies on ability to attend to a mental imagery were examined [49]. Participants who were instructed to accept tinnitus were able to focus for a longer period of time on the imagery than participants in a control condition (no instructions), but no significant difference was observed between the suppression and acceptance condition.

To indirectly test control strategies in tinnitus perception, Hesser et al. [50] manipulated ability to control background sounds in participants with tinnitus. Control was associated with a rebound effect over time, that is, participants who were instructed to control the background sounds (an active choice of type and loudness) exhibited an increase in self-rated tinnitus interference and a slower rate of improvement on cognitive performance measures over repeated trials when compared with participants who had no control of the backgrounds sounds (i.e., they were not able to chose background sound). Interestingly, control was initially (the first trial after manipulation) associated with lower rating of tinnitus interference, suggesting the importance of examining temporal aspects of the effects of different control strategies. Indeed, this finding parallel that people can in fact successfully suppress thoughts, but the detrimental effects of suppression often occur over an extended period when the thoughts rebound [2]. Furthermore, as noted earlier, findings suggest differential advantages of certain mental strategies (monitoring, distraction) depending on the length of stimulus presentation [31]. This may be one explanation to discrepancy in findings observed in experimental studies that have examined the effects of control or suppression in participants with tinnitus. That is, given that suppression may cause effects of significant magnitude over time (i.e., postsuppression rebound effect), future experiments on suppression (or related strategies) need to employ a design that will allow to examine the delayed effects of such mental control efforts.

In the present study, we used an emotional sound to simulate tinnitus and associated affective reactions in participants without auditory deficits. Tinnitus can be qualitatively similar to externally generated sounds [8]. In other words, the tinnitus sound can at least partly be described by using audiological equipment and synthesizers. Given this, a few studies [51], [52] have simulated the experience of tinnitus in laboratory settings. Simulating tinnitus allows for experimental control of the auditory stimuli and eliminates influence of extraneous factors commonly encountered in experiments with participants with significant tinnitus (i.e., individual differences in affective, cognitive or audiological problems).

In the current laboratory study, we developed a model to test the unique effects of different strategies (suppression and mindfulness) that represent the opposite of two theorized aspects of mental control [3], [4]. Specifically, we examined *a*) the potential delayed costs of suppressing an affectively negative sound that mimicked the characteristics of tinnitus. We also examined whether *b*) a mindfulness induction could counteract the hypothesized detrimental effects associated with auditory suppression. To examine behavioral consequences over time, the experiment was divided into two distinct phases. Given prior research on the effects of suppression of thoughts and emotion and initial work on attentional strategies in the auditory domain (i.e., tinnitus), we predicted that the effects of suppression of an affectively negative sound would be observed in subsequent rather than concurrent task performance. In accordance with theory and research reviewed above, we also predicted that these delayed effects of suppression would be attenuated with a brief mindfulness exercise.

## Methods

### Ethics Statement

The experiment followed the ethical principles as outlined in the Declaration of Helsinki for human studies. Signed informed consent was obtained from all participants. Data were stored anonymously. The study protocol was approved by the institutional review board at Linköping University, Sweden.

### Participants

One hundred twenty-one volunteers (73 women, 48 men) were recruited from a Swedish student population. Participants age range from 18 to 37 years (*M* = 24.9, *SD*  = 3.3). Prior to taking part of the experiment, participants reported that they had no problems with tinnitus, vision, hearing, or sensitivity to noise. After completing the experiment, which lasted for approximately 20 minutes, participants were thanked, debriefed, and compensated with an USB-flash drive (cost ≈ $15 each). We excluded one participant who acknowledged that she did not comply with experimental instructions, and one participant who had clear problems with communication/comprehension, resulting in a total sample of 119 participants.

### Overall Design

To address the specific hypotheses posed in the current study, we adopted the dual-task paradigm, an experimental paradigm commonly used to test delayed effects of suppression of thoughts and feelings [25], [26]. That is, adopting this paradigm, studies have consistently shown that deliberate attempts to suppress thoughts or feelings impair performance on subsequent tasks requiring high-degree of self-control. The current experiment was designed to test the delayed effects of suppressing an affectively negative sound on persistence behavior, and to examine whether the specific effects of suppression could be reduced following a brief mindfulness induction. We used suppression of the sound, defined as a deliberate attempt to put it “out of mind” [21], [23], as the experimental manipulation.

Participants were randomly assigned to conditions of a 2 (suppression manipulation: suppression instructions vs. no instructions) ×2 (counterinduction manipulation: mindfulness vs. attention control) design. All participants performed two mental tasks in a fixed order while exposed to the auditory stimulus: They completed a memory test and then performed mental arithmetic. Manipulation of suppression was done prior to the memory test and was intended to direct participants to use the mental strategy during the first task. Manipulation of the counterinduction was done prior to the mental arithmetic task (the second mental task). After both tasks, participants responded to a trial demand questionnaire. The time participants could persist in working with mental arithmetic while being exposed to the affectively negative sound in the second task was recorded and served as the dependent variable in the experiment.

### Materials

#### Auditory stimulus

An artificial high frequency, high pitched sound (a sinusoidal tone, amplitude modulated at approximately 100 Hz, with a center frequency of 4.5 kHz) was used in the experiment. The sound was presented binaurally in the headphones (Telephonics TDH 39P) using an AD 229e diagnostic audiometer with a loudness level at ear level of 65 dB HL (hearing level). The sound has been used in a previous study in which 12 tinnitus-like sounds were developed to simulate the psychoacoustics of tinnitus [53]. These sounds have shown to elicit physiological and subjective stress responses in normal hearing participants and in participants with tinnitus [53]. To further validate the stimulus used in the present study, 10 participants not involved in the study rated the sound and 12 sounds selected from the International Affective Digitized Sounds system (IADS-2 [54]). Participants rated a total of 16 sounds: 4 tinnitus-like sounds, 4 affectively negative sounds from the IADS-2 (normative mean valence ratings below 4; sound no. 106, 115, 380, 709), 4 affectively positive sounds from the IADS-2 (normative mean valence ratings above 6; sound no. 151, 226, 251, 360), and 4 affectively neutral sounds from the IADS-2 (normative mean valence ratings between 4 and 6; sound no. 322, 403, 700, 708). Using the normative rating methods (see, [54], for a thorough description of rating procedures), these participants rated each sound based on two separate dimensions (the Valence and the Arousal dimension; each dimension rated on 9-point scale, with high ratings indicating high pleasure and high arousal, respectively). We selected 8 sounds on the endpoints of the valance dimension: 4 were affectively negative (normative mean valence ratings of 4 or lower) and 4 were affectively positive (normative mean valence ratings of 6 or higher). Sounds were presented in a random order. The auditory stimulus used in the present study was rated as significantly more unpleasant (*M* = 2.4, *SD*  = 1.7) and arousing (*M* = 7.0, *SD*  = 1.9) compared with ratings for 4 affectively positive sounds (Cohen's *d* range  = 1.72 to 2.10, and Cohen's *d* range  = 0.59 to 1.39, respectively). In addition, the sound was rated as significantly more unpleasant compared with 3 out of 4 affectively negative sounds (all Cohen's *d*>0.77).

#### Serial recall test

To induce cognitive load during the suppression manipulation, participants completed the serial recall test (adapted from Jones & Macken [1]). The serial recall test consisted of seven letters (R, H, L, K, F, M, Q). Each letter was displayed at the center of the computer screen for 1 second. After all seven letters had been displayed, participants were asked to repeat the letter sequence in the order they were presented using a keyboard. Participants had 1 minute to recall the letter sequence, after which a new trial began. After completing one practice trial, participants completed 7 trials of the test.

#### Attentional strategy manipulation

Participants randomly assigned to the suppression condition were given instructions to suppress the auditory stimulus prior to engaging in the first cognitive task. Instruction were adapted from instructions used in previous experiments on suppression of pain and thoughts [23], [55]:

“Your task is to use all your mental power to suppress the sound you're about to hear. We know this can be hard but you should still try as hard as you can to not think about the sound. If you still notice the sound then mentally block it as quickly as possible. Suppress it. Distract yourself. Fight against it and retake control!”

To further reinforce the manipulation, participants were also told to press a red button on the desk in front of them each time they noticed the sound (c.f., thought suppression; [56]). In contrast, participants in the control condition were given no such instructions.

#### Counterinduction manipulation

Following the first mental task, participants listened to a 300 seconds audio recording. Participants randomly assigned to the mindfulness induction condition listened to a brief mindfulness exercise, informed by interventions in treatments [57] and rationales used in experimental settings [3], [28], [33]. The aim of the exercise was to have participants to direct their attention towards inner experiences, in particular their breathing. Participants were asked to notice, observe and accept feelings, thoughts and sensations in the moment. Participants were also instructed to notice discomforting sensations and actively explore such sensations without struggling with them; they were, for example, told “to make room for difficult sensations” and that “Thoughts are thoughts. Physical sensations are physical sensations, and feelings are feelings. Nothing more, nothing less. Don't struggle with the discomfort, let it be there”.

Participants randomly assigned to the attention control listened to a documentary on an unrelated topic in a foreign language, but fairly similar to participants' native tongue (i.e., a Danish documentary). Participants were instructed to listen carefully as they might have to answer questions on the subject later on. Listening to Danish is demanding for most Swedish persons and hence the task could be described as being difficult. The task was used to control for attention and experimental demand (c.f., [28], [34]).

#### Trial demand questionnaire

Following the mental tasks, participants responded to four questions on trial demand characteristics concerning the following aspects: fatigue, task demand, distress caused by sound, and interference of sound. Each question was rated on a 7-point scale, with high ratings indicating greater endorsement of effect on the specific item (i.e., high fatigue, high task demand, high distress, and high interference). Due to a technical error, responses on trial demand questions were not recorded in seven instances. In those instances, responses were omitted from the analysis.

#### Dependent variable: Persistence in the context of affectively negative sound

The length of time participants worked with mental arithmetic in the presence of the affectively negative sound in the second part of the experiment served as measure of persistence in goal-directed behavior. The disruption of mental performance by task-irrelevant sound is a well-established phenomenon [1], [58]. Thus, we presumed that ignoring an affectively negative sound in this context would require high amount of effortful persistence. Mental arithmetic was used to create a laboratory analog of a series of daily mental activities/stressors that people with tinnitus often complain about (e.g., reading or writing; e.g., [9]). Furthermore, time of persistence in mentally challenging or unsolvable tasks has been frequently used as a dependent variable in studies that have experimentally manipulated self-control in dual-task paradigms [26], including studies that have employed suppression of emotion or thought to impair later attempts of self-control [25], [59], [60]. In addition, persistence at mentally challenging tasks has been used as a measure of frustration tolerance in experiments designed to examine the adverse postadaptive effects following repeatedly presented aversive noise [61].

### Procedure

Two male experimenters conducted the experiment. Participants were asked to take part in two different studies; the first examining the relationship between interfering sound and memory, and the second examining the relationship between interfering sound and mental arithmetic.

After receiving general instructions by the experimenter and completing the consent form, participants were asked to take a seat in front of a 17 inch LCD computer screen, put on a pair of headphones, and to watch the screen for further instructions. From that point on, the experimental procedures were fully automated. All instructions and visual stimuli were presented with E-prime (Version 2.0); responses and duration (ms) were automatically recorded.

Next, participants completed the serial recall test. Participants completed 7 trials of the test while being exposed to the sound presented binaurally in the headphones. Participants in the suppression condition were additionally instructed to actively suppress the sound while performing the task. Once the serial recall test finished, the sound stopped, and all participants responded to the questionnaire on trial demand characteristics. Next, all participants listened to an audio recording (300 s). Half of the participants listened to a brief mindfulness exercise (counterinduction condition), whereas the rest of the participants listened to the documentary (attention control condition).

Following the mindfulness induction or the control task, all participants completed math assignments (1-by-1, 2-by-2, and 3-by-3 digit multiplication tasks presented in a fixed order) while exposed to the same affectively negative sound used during the serial recall test. Each assignment was displayed at the center of the screen and participants used the keyboard to answer. Participants were told that there were an infinite number of math assignments and that they were to solve as many math assignments as possible, but to stop when they wanted to give up. After each assignment, participants responded by pushing a marked key to either continue to the next assignment or stop. No participant was, however, allowed to exceed 20 minutes on this task. After giving up, participants answered the questionnaire on trial demand characteristics. The length of time (ms) from the first stimulus (i.e., first math assignment) until the participant voluntarily ended the task was recorded.

### Statistical Analysis

Before conducting the primary analysis, data were checked for significant outliers, missing cases, data analytical and distributional assumptions. The main dependent variable in the experiment (i.e., time of persistence in the second task) was analyzed using a 2 (suppression manipulation: suppression instructions vs. no instructions) ×2 (counterinduction manipulation: mindfulness vs. attention control) analysis of variance (ANOVA). Congruent with the hypothesis that the delayed effects of suppression would differ as a function of counterinduction manipulation, we predicted to observe a significant two-way interaction between suppression and counterinduction manipulation. Specifically, we expected to observe a pattern of results to support that, in the attention control, participants who were instructed to suppress the sound in the first task (the serial recall task) would on average persist for a shorter amount of time than participants who did not receive such instructions. In contrast, we expected that, in the mindfulness condition, participants who were instructed to suppress would on average persist for a similar amount of time as participants who did not receive such instructions. To further examine the interaction, simple planned comparisons (uncorrected *t*-tests) were made between suppression and no suppression instructions within each induction (i.e., mindfulness and attention control). Assuming an alpha level of .05, the current sample size provided the primary analysis with sufficient statistical power (1 – *β*  = .80) to detect a moderate effect size.

## Results

### Immediate Effects of Auditory Suppression

Participants made an average of 29.3 (*SD*  = 8.1) correct responses on the serial recall test across the seven trials. However, participants in the two conditions (suppression instruction vs. no instruction) did not significantly differ on the mean number of accurate responses on the serial recall test across trials, *t*(117)  = 1.14, *p* = .25. Similar, average subjective ratings of trial demand characteristics did not differ as a function of condition (all *t*'s <1.34; all *p*'s >.19). Means (with standard deviations in parentheses) for experimental demand questions fatigue, task demand, distress of sound, and interference of sound were 3.2 (1.4), 3.6 (1.6), 4.6 (1.4), and 3.9 (1.7), respectively.

### Delayed Effects: Persistence in the Context of Interfering Emotional Sound

**Figure 1:**
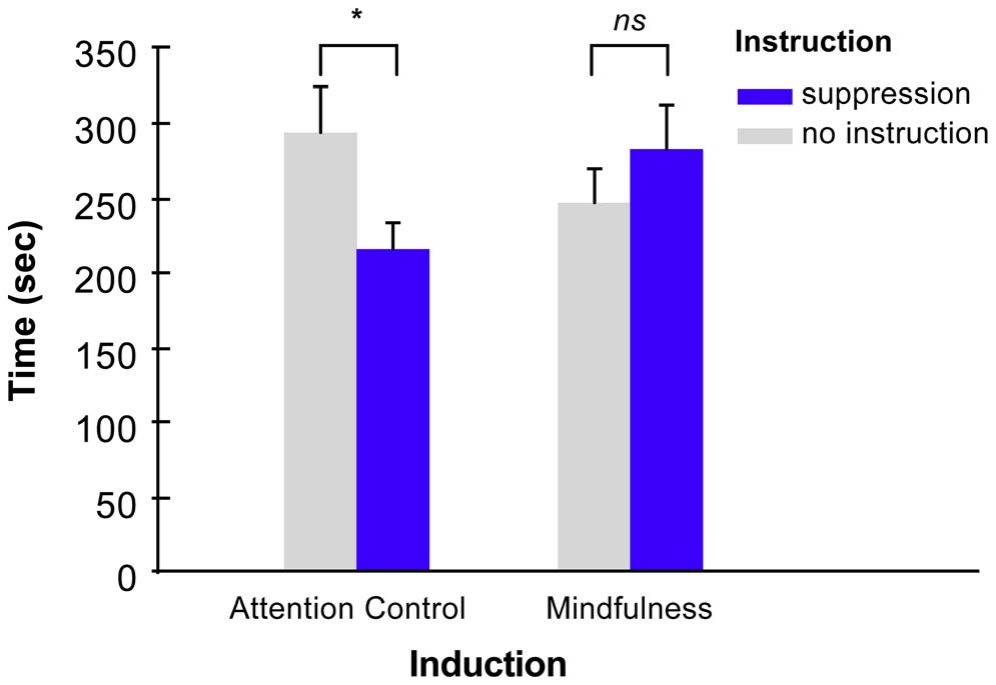
Time of effortful persistence as function of experimental manipulations (instruction and induction). Participants were required to ignore emotional task-irrelevant sound while performing a mentally challenging task (mental arithmetic). Induction manipulation (attention control vs. mindfulness induction) followed the suppression manipulation (suppression vs. no instructions) in the experiment; thus, these are the delayed effects of suppression. Error bar denotes standard error of mean. *  =  *p*<.05; *ns*  =  non-significant at specified alpha level (.05).

The length of time spent attempting to solve math assignments in the subsequent task served as measure of persistence in goal-directed behavior in the presence of the affectively negative sound. Participants could on average persist in the task for 260 seconds (*SD*  = 140). Our hypotheses predicted that suppression of the sound during the first task would produce reduced persistence, but that a mindfulness induction between tasks will attenuate this effect. Figure 1 depicts means as a function of manipulations. A 2 (suppression manipulation: suppression instructions vs. no instructions) ×2 (counterinduction manipulation: mindfulness vs. attention control) analysis of variance (ANOVA) revealed neither a main effect of suppression, *F*(1, 115)  = 0.68, *p* = .41, η^2^ = .01, nor of counterinduction, *F*(1, 115)  = 0.16, *p* = .69, η^2^ = .00. However, as predicted, there was a statistically significant interaction effect of suppression × counterinduction, *F*(1, 115)  = 5.12, *p* = .026, η^2^ = .04. In the attention control condition, participants who received instructions to suppress the sound in the first task (during the serial recall test) spent significantly less time persisting (*M* = 216 s, *SD*  = 95, *n* = 29) than those who received no such instructions (*M* = 294 s, *SD*  = 168, *n* = 30), *t*(57)  = 2.18, *p* = .01 (one-tailed), *d* = −0.57. Thus, suppression was associated with a delayed cost. In contrast, in the mindfulness condition, participants who received instructions to suppress the sound in the first task did not spend significantly less time persisting (*M* = 283 s, *SD*  = 161, *n* = 30) than those who received no suppression instructions (*M* = 247 s, *SD*  = 109, *n* = 30), *t*(58)  = 0.31, *p* = .31, *d*  = 0.26. Thus, the mindfulness induction between tasks counteracted the detrimental effects of suppression (as illustrated in Figure 1).

Because the variances were not equal across conditions and there were some tendencies that time deviated from a normal distribution, we recalculated the main analysis with a robust estimation method (i.e., bootstrapping). The results remained the same. In addition, *p*-values corrected for homogeneity of variance violations did not differ substantially from those reported above.

### Control Analyses

Demographic variables (age, gender) were unrelated to the dependent variable (i.e., time of effortful persistence) used in the study. To be sure that there were no time-accuracy trade-offs on the second task, such that, for example, no instruction participants in the attention control condition could persist for a longer time because they provided more correct responses on the math assignments, we included an individual's percentage of correct responses (correct response/number of assignment completed) as a covariate in the ANOVA. This did not alter the findings, that is, the interaction effect was still significant, *F*(1, 114)  = 4.96, *p* = .028. It is plausible that differences observed are a function of perceived experimental demand in the first task (fatigue, task demand, distress of sound, and interference of sound). However, correlations between experimental demand ratings from the first task and time of persistence in the subsequent task were all nonsignificant (all *r*'s <.17; all *p*'s >.05), with the exception for distress of sound that had a small negative correlation with the dependent variable (*r* = −.27, *p*<.01). Again, the interaction effect between suppression and counterinduction on time of persistence remained significant even when controlling for distress of sound, *F*(1, 114)  = 4.53, *p* = .035.

Furthermore, a series of 2×2 ANOVAs revealed no significant interaction effect on any of the mean subjective ratings of experimental demand following the second task (all *F*'s <.51, all *p*'s >.82). Taken together, this indicated that the experimental effects were not simply due to trial demand characteristics. Means (with standard deviations in parentheses) for experimental demand questions fatigue, task demand, distress of sound, and interference of sound were 4.2 (1.5), 4.3 (1.8), 5.3 (1.1), and 4.1 (1.7), respectively. Noteworthy, is that participants on average rated the sound as significantly more distressing and interfering in the second task (*M* = 4.3, *SD*  = 1.8, and *M* = 5.3, *SD*  = 1.1, respectively) than in the first task (*M* = 3.6, *SD*  = 1.6, and *M* = 3.9 *SD*  = 1.8, respectively), *t*'s >4.80, *p*'s <.001. This suggested that participants had not habituated to the auditory stimulus that was used throughout the experiment.

## Discussion

The present study examined the effects of attentional strategies in the context of an emotional interfering sound that mimicked the acoustic characteristics of tinnitus. We tested a model whereby we could study two strategies that represent the opposite of two theorized aspects of mental control. Specifically, we predicted that suppression of the auditory stimulus would result in delayed costs in the form of decreased persistence in a subsequent task that required participants to work with mental arithmetic while presented with the same sound. We also expected that a brief mindfulness induction between tasks would attenuate the delayed effects of suppression. As hypothesized, in the attention control, participants who were instructed to suppress the sound under mental load were less likely to persist in a subsequent task relative to participants who had received no such instructions. Furthermore, a mindfulness induction between tasks counteracted this effect. The significance of the latter finding is emphasized by the fact that the mindfulness exercise was brief (300 sec) and gave no specific advice on how to approach stimuli in the second task. Thus, the positive effects of the mindfulness induction are particularly noteworthy (c.f., [33], [34]). Furthermore, there was little evidence to support that these effects were due to experimental demand. The findings are broadly consistent with the accumulating body of experimental work on the impact of suppression and mindfulness/acceptance on performance and affective responses in the context of aversive stimuli in non-auditory domains.

### Attentional Strategies and Persistence in the Context of Interfering Emotional Sound

Why might deliberate efforts of trying to suppress an emotional interfering sound impair ability to persist in task-relevant behavior in the context of a subsequent presentation of the same sound, and why might mindfulness eliminate these impairments? Critical to any adaptive regulation is our ability to exert control over attention processes. Attempts to suppress a given stimulus may create paradoxical effects producing more of the suppressed targets. Suppressing an emotion stimulus (e.g., anxious thoughts) may even exacerbate the associated emotional responses [22]. Thus, deliberate efforts of keeping emotional sounds “out of mind” could potentially create a rebound effect with increased interference and negative affect over time. It is important to note that the effects of suppression were delayed rather than immediate. In fact, analyses revealed no evidence for any difference between suppression and no instruction on concurrent task performance (on the serial recall test). Indeed, these results parallel the findings from research on suppression in other domains (i.e., postsuppression rebound effect [2], [22]). For example, in the physical domain, Cioffi and Holloway [23] in a study found no difference between suppression and other attentional strategies (monitoring, distraction) on tolerance time for acute pain experience (on a cold-pressor task). Differences were, however, observed on post pain recovery ratings and on the interpretation of a subsequent somatic stimulus. The current findings extend the evidence for such postsuppression rebound effects by suggesting divergent effects of auditory suppression depending on time of assessment (i.e., immediate vs. delayed).

Wegner's [2], [22] theory on cognitive processes involved in thought suppression may provide a useful explanation for the delayed effects observed. The theory posits that there are two distinct processes that operate over different temporal periods: An intentional operating process that seeks to produce the desired mental state by using distractors, and an involuntary ironic process that actively searches for the to-be-suppressed target. First, during suppression, the ironic process works together with the operating process to achieve mental control, as the former signals the latter of the need to reactivate distraction efforts when conscious awareness of the unwanted mental event becomes imminent. In a later stage, however, when the operating process is voluntary terminated, the ironic process may continue to seek for the unwanted mental event, thereby creating a postsuppression rebound effect. Indeed, studies using functional magnetic resonance imaging have provided evidence that parallel the temporal aspects of the theory involved in attempts to suppress thoughts [62]. Accordingly, one explanation to impaired persistence among participants who initially engaged in suppression might be impaired attention control following an ineffective mental control strategy. That is, participants might have become more sensitive to the unwanted sound in the second task due to a lingering monitoring process of the sensation, making them less likely to continue with the task in the presence of the sound.

The mindfulness induction reversed the effects of suppression: Participants who initially were instructed to suppress the sound but later received a brief mindfulness exercise were able to persist to similar extent as those who had not received instructions to suppress. What is important to note is that mindfulness did not seem to promote a general ability to persist in the second task as no significant main effect of induction was found in the current experiment; rather, the effects of mindfulness seem to be specific. This finding is highly consistent with theories that posit that mindfulness and related processes such acceptance may undermine ineffective control strategies [35], [36]. Wegner [4] has suggested that ironic processes involved in mental control might be prevented by paradoxical interventions, mindfulness and acceptance-based techniques. This may obstruct ironic processes by providing an incompatible mental approach to mental control strategies such as suppression. Thus, promoting people to attend mindfully to sensations may prevent them from using ineffective strategies (i.e., suppression). Furthermore, acceptance of an uncontrollable sound and associated emotional responses – noticing and embracing them without resisting them – may facilitate a context in which one's attention can be focused on the primary task irrespectively of interfering irrelevant stimuli, thereby, minimizing interference and demand. Indeed, preliminary evidence support that mindfulness training may achieve its positive effects through enhanced working memory capacity, response inhibition and executive control [63], [64]. Furthermore, acceptance has been found to be more efficient in terms of resources than suppression when applied to emotions [39]. This may be a product of acceptance being a non-goal oriented process; unlike suppression where the goal is to have an “empty” mind in relation to the target-to-be suppressed sensation, acceptance as a mental strategy desires no specific mental end-state. As such, it may require fewer resources and therefore offer greater opportunities for flexible interactions with the environment [37], [39]. Still, the discussion above is only speculative and future work should try to illuminate the processes involved in the phenomena that were observed here. Further investigations should focus on how suppression of auditory stimuli may disrupt mental activities and how mindfulness may work to undermine potentially negative effects of auditory suppression.

### Implications for Tinnitus

Given that we simulated tinnitus in the current experiment, our results may only have limited relevance for the understanding of tinnitus. It could be argued, of course, that the very nature of the experiment (i.e., individuals exposed to an affectively negative sound) falls short of providing an explanation for tinnitus distress and interference. Notwithstanding this, our findings are broadly consistent with work showing that avoidance-based coping is associated with increased tinnitus-related distress [43] and that acceptance-based techniques, including mindfulness, can alleviate suffering caused by tinnitus [45]. It is plausible that specific mental efforts to control the experience of tinnitus can work temporarily, reducing thoughts about tinnitus [48] or interference of the sound [50]. However, such strategies may not work over time when it comes to an uncontrollable auditory perceptual phenomenon such as tinnitus [50]. Indeed, our results provide initial support to the notion that suppressing a tinnitus-like sound can be associated with delayed costs.

How an individual attend, process and evaluate tinnitus may play an important part in determining degree of tinnitus impact [18]. Yet, the mechanisms involved in tinnitus distress are still poorly understood. It has been suggested that cognitive interference may act as starting point for later evaluative emotional processes in tinnitus [17]. The present results extend such a theoretical model by focusing on how a specific attentional control strategy may influence the ability to engage in mental activities over time in the context of emotional interfering sound. That is, individuals who engage in suppression of tinnitus may experience problems with sustained mental control in everyday activities such as reading, writing or listening. This may cause a vicious circle in which tinnitus becomes associated with negative emotional reactions, which in turn, results in increased attention to tinnitus and cognitive interference. Approaching and paying attention in a non-evaluative way to tinnitus might be one way to undermine the natural tendency to suppress, and thus, preventing a preoccupation with the sensation. In fact, the ability to accept, notice and distance oneself from inner sensations associated with tinnitus has been related to improved long-term outcomes in acceptance-based treatment [42]. Yet, a considerable amount of more work is needed to explore the psychological underpinnings of such a cycle.

### Limitations

Although the present results are promising, the study has limitations. First, the study relied on simulated tinnitus. Although previous research has been able to mimic the characteristics of tinnitus, both in terms of psychoacoustics and affective responses [51], [52], the results of our study may only with caution be applied to tinnitus. That is, there might be significant differences between tinnitus, which is the experience of sound in the absence of any appropriate external source, and externally generated sounds with regard to the psychological processes involved in sound perception and interference. Nevertheless, we believe that simulated tinnitus in laboratory settings offers a unique opportunity to explore mechanisms involved in both development and treatment of tinnitus distress. Such experiments allow for greater experimental control of auditory stimuli and eliminate extraneous factors commonly encountered in experiments with participants with significant tinnitus (i.e., individual differences in affective, cognitive or audiological problems).

Second, participants were recruited from a student population. Thus, the findings may not generalize to other non-clinical and clinical populations. Third, we do not know the extent to which participants followed the scripted instructions to suppress the sound. On the other hand, it is difficult to conduct appropriate manipulation checks of mental control strategies and most studies that have examined suppression in other domains have relied on self-report to assess compliance with the mental strategy employed [19]. Still, future studies should develop and include objective measures to assess both compliance with mental instructions and cognitive accessibility of the avoided sensation, similar to the work that has been done on thought suppression [22].

Fourth, it is, of course, important to remember that the effects of the suppression manipulation cannot be interpreted in isolation from the second manipulation in the experiment (i.e., induction manipulation). Thus, future studies should examine the effects of auditory suppression in other experimental paradigms (e.g., without using any induction between tasks) and extend the work by systematically varying cognitive load, emotional valence of the sound, and by using other dependent measures of relevance to the phenomenon (subjective loudness of the sound, physiological states, behavioral avoidance in the context of aversive auditory stimuli, cognitive performance measures etc.).

Fifth, the ability to generalize effects of the intervention to a clinical setting is limited given the short mindfulness exercise that was used in the current experiment. Indeed, it has been argued that a “true” mindfulness induction requires extensive training [33] and in clinical practice, mindfulness is often promoted over weeks of training. On the other hand, positive effects of brief laboratory manipulations of mindfulness-related processes have been shown. For example, Erisman and Roemer [34] demonstrated that a brief mindfulness-type intervention (10 minute) could positively influence affective reactions.

### Conclusions

Our work developed and tested a theoretical model based on how mental approaches may affect ability in the context of task-irrelevant emotional sound. To our knowledge, it is the first study of its kind. We demonstrate a novel phenomenon in which effortful suppression of an emotional sound can be associated with delayed costs on performance in the context of task-irrelevant sound, and that experimentally induced mindfulness can reduce the detrimental effect of auditory suppression. We encourage further investigations to continue this line of research. Such research efforts may not only have implications for tinnitus, but more broadly be relevant to situations in which unchangeable sound intrudes and disrupts performance, a common phenomenon in everyday life [58].
